# Cognitive control and the non-conscious regulation of health behavior

**DOI:** 10.3389/fnhum.2015.00122

**Published:** 2015-03-05

**Authors:** Amanda L. Rebar, Andrea M. Loftus, Martin S. Hagger

**Affiliations:** ^1^School of Human, Health, and Social Sciences, Central Queensland UniversityRockhampton, QLD, Australia; ^2^Curtin Neuroscience Laboratory, School of Psychology and Speech Pathology, Curtin UniversityPerth, WA, Australia; ^3^Health Psychology and Behavioural Medicine Research Group, Laboratory of Self-Regulation, School of Psychology and Speech Pathology, Curtin UniversityPerth, WA, Australia

**Keywords:** automaticity, motivation, physical activity, habit, implicit

We agree with Buckley et al. (2014) that self-control processes are one important aspect of physical activity and sedentary behavior regulation, and that self-control training is an important avenue for health behavior intervention research. However, we believe the role of non-conscious regulatory processes of health behaviors was understated in that the focus was mostly on how non-conscious temptations can bias one toward unhealthy behaviors. We take this opportunity to extend this discussion by highlighting that health behaviors are also regulated by non-conscious processes, and that cognitive control training may also work to regulate behavior through these regulatory pathways.

Buckley and colleagues propose that cognitive control abilities are instrumental for the regulation of physical activity, and this is supported by decades of accumulated evidence of the influence of self-regulation processes (e.g., goals, intentions, planning). However, this evidence shows that, at most, half of the variability of physical activity is explained by these constructs (e.g., Brassington et al., 2002; Webb and Sheeran, 2006; Rhodes and Dickau, 2012) suggesting a equally important role of other pathways to physical activity participation and persistence, of which non-conscious pathways are likely to be strong candidates.

Health behavior is also driven by *non-conscious processes* that predispose individuals to act and are the manifestations of well-learned cue-response pairings (Dimmock and Banting, 2009; Rothman et al., 2009; Orbell and Verplanken, 2010; Marteau et al., 2012; Sheeran et al., 2013; Grove et al., 2014; Hagger et al., 2014). In terms of proposed mechanisms, non-conscious processes are related to physical activity by eliciting affective and visceral responses that occur within a fraction of a second after cue perception preceding any controlled deliberation (Murphy and Zajonc, 1993; Bargh et al., 1996; Cunningham et al., 2007). This is not to say that we think physical activity is exclusively determined by non-conscious processes. On the contrary, we propose that physical activity is a complex behavior determined by an interaction of the two. For example, an individual may make a quick, spontaneous, non-deliberative decision to exercise on the basis of the presentation of a well-learned cue (e.g., viewing their exercise program on the wall upon waking), but the knock-on effect of the decision may bring multiple well-learned but consciously-directed decisions into play (e.g., deciding to run or swim, choosing to do it alone or with others). So a non-conscious process may set in motion a series of more consciously-controlled processes leading to action. Attraction and approach responses to physical activity are an important consideration when predicting and understanding physical activity behavior, as outlined by the correlations observed between decision tasks containing activity-related stimuli and physical activity participation (Conroy et al., 2010; Hyde et al., 2012).

Research in neuroscience supports the contention that behaviors like physical activity are not exclusively the result of conscious processes and can, to some extent, become guided by automatic processes. Physical activity is often considered to be controlled by deliberative pathways, with concomitant activity in the frontal-parietal and cingulo-oppercular networks. However, subcortical areas of the mesolimbic reward system, which represent reward and emotional valence of stimuli, including the nucleus accumbens, are also activated when individuals engage in acts of self-regulation (Heatherton and Wagner, 2011; Hagger and Chatzisarantis, 2013). This reward system, which likely works outside of a person's conscious awareness (Cunningham et al., 2004), can become trained to respond to certain cues based on learned reward expectancies, and so become hyperactive when salient cues or stimuli are present. For example, repeated presentation of stimuli that are initially controlled by deliberative pathways within the frontal parietal network may accompany feedback from the dopaminergic pathways in the subcortex, such as the mesolimbic dopamine system. Over time, the intrinsic rewards develop and lead to strong reinforced pathways to action. For example, in the early stages of adopting a behavior like physical activity, engagement of the behavior may initially be regulated through conscious control and determined by deliberative pathways, but concomitant responses in the subcortical reward regions of the brain in response to physical activity may reinforce the pathways and compel an individual to return to the activity. After sufficient repetition, the process becomes less deliberative, and the pathways determining the initiation of the action in response to salient cues are met with stronger neural activity relative to competing processes, such that the decision-making processes leading to action are strong and efficient (Miller and Cohen, 2001; Heatherton and Wagner, 2011; Labrecque and Wood, 2015).

As highlighted by Buckley et al. (p. 3), there are costs to overexertion of self-regulation, and individuals tend to be confronted with an array of competing demands and alternative goals (Hagger, 2013; Kurzban et al., 2013). We propose that using self-control training, referred to as cognitive control training by Buckley and co-authors, can also enhance the non-conscious regulation of health behavior. Training may be more beneficial for health behavior maintenance than strictly focusing on the enhancement of consciously regulated processes. People may be more effective at maintaining healthy behaviors by shifting more behavior regulation over to non-conscious processes, thereby reducing the need to consciously attend to these processes. A meta-analysis of the effects of self-control on a wide range of behaviors showed that self-control is more strongly linked to non-conscious behaviors than to consciously-regulated behaviors (De Ridder et al., 2012), which highlights the potential for the utilization of self-control training as a means to enhance non-conscious regulation of physical activity via the formation and maintenance of strong habits. This evidence supports the proposition put forth by Hagger and colleagues (Hagger and Chatzisarantis, 2014; Hagger and Luszczynska, 2014) that self-control can also act on behavior through non-conscious means.

In summary, we contend that decisions to engage in physical activity may be partially determined by non-conscious processes. This pathway has tended to been neglected in previous research and was not explicitly outlined by Buckley et al. We agree with Buckley et al. that deliberative conscious control is required to overcome the powerful well-learned pathways to sedentary behavior and compete with weaker, less well-defined pathways leading to decisions to be physically active. Together, these processes form part of a holistic approach to understanding neural pathways to physical activity.

## Conflict of interest statement

The authors declare that the research was conducted in the absence of any commercial or financial relationships that could be construed as a potential conflict of interest.
